# Prevalence and Risk Factors for Anemia in a Population With Hidradenitis Suppurativa

**DOI:** 10.7759/cureus.12015

**Published:** 2020-12-10

**Authors:** Sydney R Resnik, Emily L Geisler, Nicolas Reyes, Juan Lozano, Rafael A Ramirez-Caussade, Barry Resnik

**Affiliations:** 1 Department of Translational Medicine, Florida International University, Herbert Wertheim College of Medicine, Miami, USA; 2 Department of Internal Medicine, HCA Healthcare/University of South Florida Morsani College of Medicine, Blake Medical Center, Bradenton, USA; 3 Department of Anesthesiology, Riverside Hampton Surgical Center, North American Partners of Anesthesia, Hampton, USA; 4 Department of Dermatology, Dr. Phillip Frost Department of Dermatology & Cutaneous Surgery, University of Miami Miller School of Medicine, Miami, USA; 5 Department of Dermatology, Resnik Skin Institute, Aventura, USA

**Keywords:** anemia, prevalence, hidradenitis suppurativa

## Abstract

Background: Hidradenitis suppurativa (HS) is an inflammatory disease presenting as nodules evolving into scarred plaques. HS is associated with many co-morbidities, including anemia of chronic disease, though few studies report on this association.

Objectives: This study evaluated the prevalence of anemia among the HS patient population and potential associations between risk factors for HS and anemia development.

Methods: Records diagnosed HS patients in one private practice (BR) were reviewed by investigators. The 92-patient cohort was evaluated for multiple data-points and responses grouped based on age, gender, ethnicity, body mass index (BMI), smoking status, and comorbidities. Data were analyzed using STATA to perform descriptive analysis and bivariate analysis.

Results: The prevalence of anemia in this cohort was 41.3%. Of anemic patients, the majority were men (65.2%), African American (60.6%), and never/former smokers (48.6%). There was a significant increase in the odds of developing anemia in HS patients that are men (odds ratio (OR) 3.8) and African American (OR 3.5).

Conclusions: We show that the prevalence of anemia in an HS patient population greatly surpasses that of the U.S. population (~5%). It is clear that anemia is a significant complication for HS patients. We hope that physicians can recognize the importance of screening patients with HS for anemia to medically optimize treatment for their patients.

## Introduction

Hidradenitis suppurativa (HS), also known as acne inversa, is a dermatologic disease that presents as tender, subcutaneous nodules, which may spontaneously rupture and coalesce into scarred plaques and deep dermal abscesses [1]. For many years, HS was thought to be a disorder of apocrine origin, but it is now generally agreed that occlusion of the hair follicle is the first step in the disease process. These follicles can then rupture and extrude their contents, including bacteria, sebum products, and hair, into the dermis, creating a chemotactic inflammatory response [1]. This is followed by an inflow of neutrophils, lymphocytes, and histiocytes, which leads to abscess formation and infection with suppuration (pus formation) and release of serous to purulent malodorous discharge. With chronic recurrence, sinuous tracts, fibrosis, dermal contractures, and induration of the skin occur [1].

HS typically presents after puberty; the average age of onset of HS is in the second to third decades of life. The prevalence of HS may diminish over time, as one study showed that prevalence among those 55 years and older was significantly lower than in younger age groups (0.5% vs 1.4%) [1]. Prevalence reports for HS range from 0.3% to 4%, however, the currently agreed upon the global prevalence of HS is 1% [2]. HS is generally accepted as more common in women, with a female:male ratio of approximately 3:1 [2]. Some researchers argue that the location of involvement appears to have a sexual predilection, as perianal HS seems to affect men more than women [1]. Some factors associated with HS are smoking and obesity. Studies have shown an increased prevalence of smoking among patients with HS, with some authors suggesting that tobacco may be a potential trigger for HS [1]. In another study looking at obesity and HS, 51.6% of subjects with HS were obese, and 21.5% were markedly obese. Obesity is felt to be an aggravating factor in this disease through sweat retention and shearing of follicular and ductal outlets [1].

HS is one of four diseases of a group called follicular occlusion or acne tetrad. The other conditions in the tetrad are acne conglobata, pilonidal cyst, and dissecting cellulitis [3]. These diseases are often diagnosed with the same patient and may occur simultaneously or at different times throughout the patient's lifetime. Acne conglobata and pilonidal cysts appear to be the most commonly linked conditions with HS [3]. Other comorbid conditions found in HS patients include several types of arthritis, such as reactive inflammatory arthritis, spondyloarthropathy, systemic lupus erythematosus, and pyoderma gangrenosum [3]. HS patients can also present with many different complications in the course of their treatment. These can include contractures and limb mobility limitations due to fibrosis of the abscess tracts, metabolic syndrome, and anemia [3]. Anemia is of most interest in the current study and tends to be overlooked in this patient population, as evidenced by a paucity of studies reporting on the association between HS and anemia [3]. Furthermore, it remains uncertain if anemia in these patients is anemia of chronic disease or if there are other factors at play. 

Anemia is defined as a decreased hemoglobin level in the blood, less than 13.0 grams per deciliter (g/dl) for males and less than 12.0 g/dl for females [4]. There are several types of anemia, and they are categorized based on the size of the erythrocyte upon microscopic examination. Multiple factors play a role in the development of anemia, including diet, weight, social habits, and disease states. Anemia of chronic disease occurs as a result of a chronic or acute inflammatory/immune process [4]. The prevalence of anemia of chronic disease is second only to anemia due to iron deficiency. The genesis of anemia of chronic disease begins with the cytokines released during an inflammatory response. This affects the levels of iron, transferrin, and ferritin in the body. The inflammatory cytokines cause the body to decrease iron absorption via the production of hepcidin and promote the uptake of iron into cells for storage as ferritin [4]. This results in a decreased availability of iron for the hematopoietic system leading to the development of normochromic, normocytic anemia [4].

Although HS is a disease involving a heavy inflammatory response, there is still some speculation regarding the co-occurrence of anemia and HS. A 1968 study of 42 consecutive cases of HS showed that nearly twenty-four percent had marked anemia with hemoglobin levels below 10.0 g/dl. In each of these cases, the patients had severe anemia for more than two years. The authors of the study ultimately hypothesized that the anemia was secondary to chronic infection [5]. On the other hand, a much larger study conducted in Denmark in 2015 concluded that there was no causal association between HS and anemia found in patients suffering from the disease [6]. In 2016, a brief editorial was published outlining the occurrence of anemia in two patients with HS [7]. In both of these cases, the HS patients presented with fatigue and were ultimately diagnosed with microcytic anemia [7]. 

An increased incidence of anemia in the population of HS patients in a South Florida Dermatology practice was noted by treating physicians, BR and RR-C, in the context of evaluating BR’s patients undergoing general anesthesia for CO2 laser surgery at an ambulatory surgical center. The current study was prompted by these observations. The aim of this study is to determine the prevalence of anemia in this patient population and compare it to that of the general population. We also sought to determine if there were any identifiable risk factors that place these patients at increased risk for developing anemia.

## Materials and methods

A list of individuals diagnosed with HS that had received or were currently receiving treatment from BR between January 1, 2013, and January 31, 2018, was provided as potential participants in the study. Electronic medical records in PowersoftMD (Data Tec Inc., Des Peres, MO) were analyzed to retrieve information regarding patient anemia status as determined by hemoglobin level, as well as risk factors evaluated in the study, including age, sex, body mass index (BMI), hypertension status, diabetes status, and smoking status. Patients were excluded if laboratory studies for anemia were not available.

Patient records were accessed through a secure browser and stored in a password-protected document to ensure HIPAA regulation and patient privacy. Upon completion of data retrieval, the information was first analyzed to determine the prevalence of anemia in the HS population. Further analysis was completed to determine the associations between each of the identifiable risk factors and anemia via bivariate analysis to check for unadjusted associations.

## Results

Population characteristics

**Figure 1:**
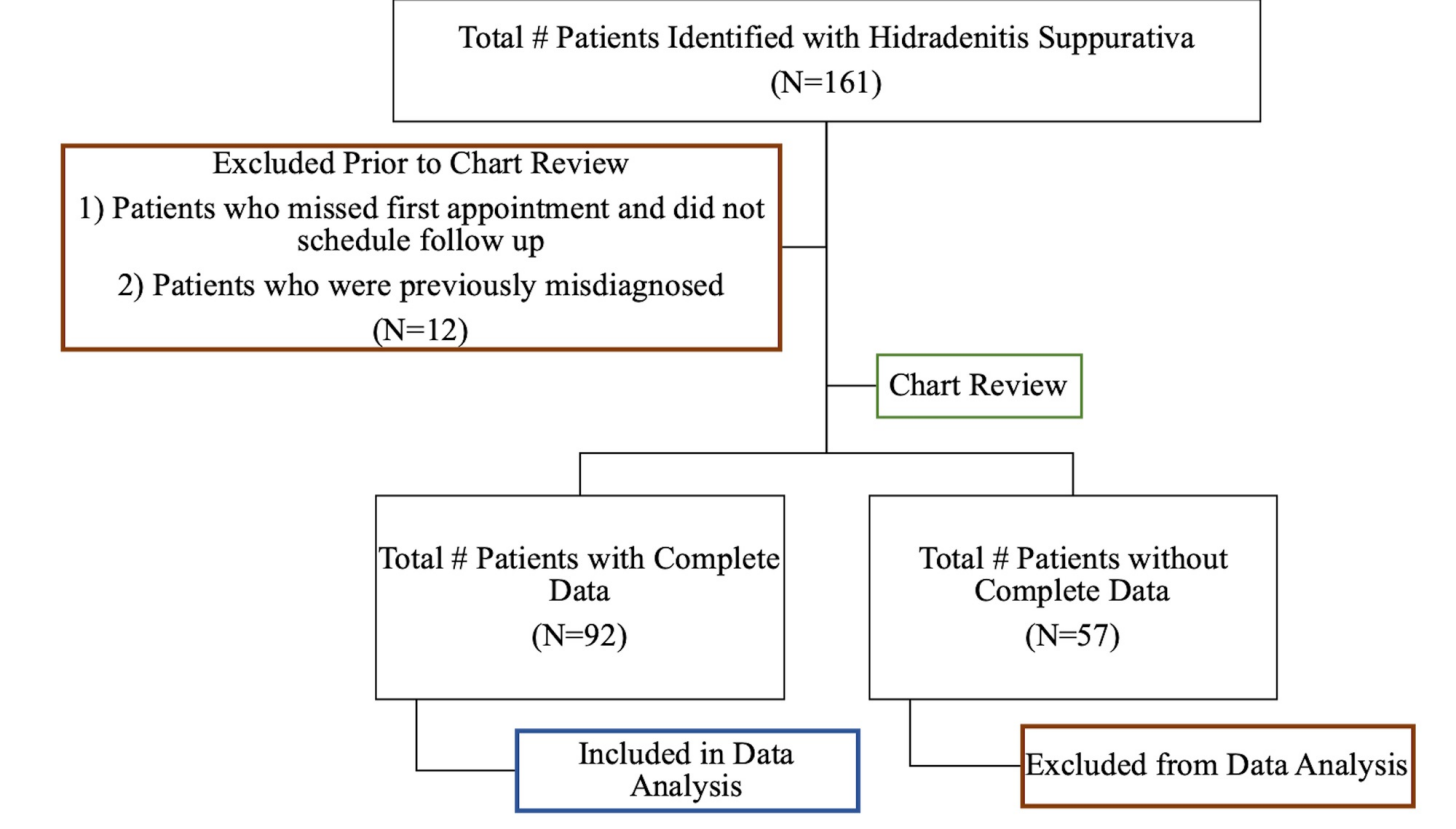
Identification of final patient population for analysis

After an initial survey of office electronic medical records, 161 patients with a diagnosis of HS were identified. Twelve patients were excluded because they missed their first appointment or because they had been previously misdiagnosed. Following chart review, 57 patients with HS were excluded from analysis due to incomplete information regarding the risk factors examined in this study (Figure 1). A comparison between included and excluded patients based on distribution age and gender was carried out and did not show a significant difference between the groups (data not shown). Data from a final total of 92 patients were analyzed (Table 1). The majority of the population was Caucasian and female. Approximately 43% of the population was 30 years or older. In our population, 94.6% were non-diabetic and 90.2% did not have hypertension. Furthermore, 80.4%, reported being former or non-smokers. Nearly 42% of participants were obese with a body mass index greater than 30. Hemoglobin levels ranged from 8.1 g/dL to 15.9 g/dL. Finally, the prevalence of anemia in the population was 41.3%. Of note, several of the patients in this population had been previously evaluated by hematology for their anemias and were found to be suffering from anemia of chronic disease.

**Table 1:** Baseline characteristics of patients with HS BMI: body mass index; HS: hidradenitis suppurativa

Characteristics	N = 92	%
Age (years)		
<30	40	43.5
30-39	27	29.3
>39	25	27.2
Gender		
Female	69	75.0
Male	23	25.0
Race		
Caucasian	39	42.4
African American	33	35.9
Hispanic/Other	20	21.7
BMI		
Non-obese (< 30.0)	27	29.4
Obese (≥30.0)	38	41.3
Not reported	27	29.4
Smoking history		
Current	18	19.6
Never/former smoker	74	80.4
Hypertension		
Yes	10	10.8
No	82	0.9
Diabetes (Type 1 & Type 2)		
Yes	5	5.4
No	87	94.6
Anemia		
Yes	38	41.3
No	54	58.7

Bivariate analysis

Next, we conducted a bivariate analysis to explore the association between the risk factors of interest and anemia (Table 2). Noticeable differences between genders were found, with approximately 65% of the male population having anemia compared to only 33% of the female population. A larger percentage of African Americans were anemic as compared to any of the other races/ethnicities in the population. A slightly larger percentage of diabetics had anemia as compared to non-diabetics (60% vs 40%). Finally, never/former smokers had a greater percentage of anemia than current smokers.

**Table 2:** Distribution of baseline characteristics in patients with HS with or without anemia* *All values are percentages. BMI: body mass index; HS: hidradenitis suppurativa

Characteristics	Anemia Status		p-Value
	Yes (N = 38)	No (N = 54)	
Age (years)			0.703
<30	37.5	62.5	
30-39	40.7	59.3	
>39	48	52	
Gender			0.007
Female	33.3	66.7	
Male	65.2	34.8	
Race			0.019
Caucasian	30.8	69.2	
African American	60.6	39.4	
Hispanic/Other	30.0	70.0	
BMI			0.194
Non-obese (< 30.0)	55.6	44.4	
Obese (≥30.0)	36.8	63.2	
Not reported	33.3	66.7	
Smoking history			0.004
Current	11.1	88.9	
Never/former smoker	48.6	51.4	
Hypertension			0.929
Yes	40.0	60.0	
No	41.5	58.5	
Diabetes (Type 1 & Type 2)			0.383
Yes	60.0	40.0	
No	40.2	59.8	

Crude and adjusted associations

Bivariate analysis was done to examine the relationship between each risk factor and its association in developing anemia in the HS patient population (Table 3). The data demonstrated that there was a statistically significant increased odds of developing anemia in male patients, African American patients, and patients who were smokers. The odds of developing anemia increased nearly four times in men when compared to women (odds ratio (OR) 3.8, 95% CI 1.4-10.1, p-value 0.009). African Americans demonstrated a three and a half times increase in the odds of developing anemia when compared to Caucasians (OR 3.5, 95% CI 1.3-9.2, p-value 0.013). Current smokers demonstrated a decrease in the odds of developing anemia (OR 0.1, 95% CI 0.0-0.6, p-value 0.01). Although not statistically significant, it was found that diabetics had a roughly two times increase in the odds of developing anemia. Obese individuals had decreased odds of developing anemia, as did patients with hypertension. Individuals age 30 or greater had a minimal increase in the odds of developing anemia when compared to those under 30 (Table 3).

**Table 3:** Unadjusted OR of anemia in patients with HS by characteristic OR: odds ratio; BMI: body mass index; HS: hidradenitis suppurativa

Characteristic	OR (95% CI)	p-Value
Age (years)		
<30	Reference	
30-39	1.1 (0.4-3.1)	0.79
>39	1.5 (0.6-4.2)	0.404
Gender		
Female	Reference	
Male	3.8 (1.4-10.1)	0.009
Race		
Caucasian	Reference	
African American	3.5 (1.3-9.2)	0.013
Hispanic/Other	1.0 (0.3-3.1)	0.952
BMI		
Non-obese (< 30.0)	Reference	
Obese (≥30.0)	0.5 (0.2-1.3)	0.137
Not reported	0.4 (0.1-1.2)	0.103
Smoking history		
Current	0.1 (0.0-0.6)	0.01
Never/former smoker	Reference	
Hypertension		
Yes	0.9 (0.3-3.6)	0.929
No	Reference	
Diabetes (Type 1 & Type 2)		
Yes	2.2 (0.4-14.0)	0.393
No	Reference	

A multivariate analysis was attempted using the risk factors that demonstrated a statistically significant difference in the odds of developing anemia. The adjusted and unadjusted values obtained during this analysis demonstrated high variability and low precision due to the small sample size of this study.

## Discussion

Hidradenitis suppurativa is a recurrent dermatologic disease involving the development of painful subcutaneous nodules and potential scarred plaque formation. An increased incidence of anemia was noted in an HS patient population in a South Florida Dermatology practice. Following concerns raised by the treating physicians (BR & RR-C), this study was designed to assess the prevalence and relationship between HS and anemia and to identify potential risk factors for the development of anemia in these patients. 

There is little research addressing anemia in the HS population. However, there are two large studies that reported conflicting results. The first, published in 1968, demonstrated an association between HS and anemia in a sample population of patients in Texas [5]. A larger study carried out in Denmark showed no statistically significant association between hemoglobin levels indicative of anemia, and HS [6]. In the Denmark study, neither the HS populations nor the control population had an incidence of anemia higher than 10%. However, this current study demonstrated that 41.3% of the HS study population had anemia. In contrast, the prevalence of anemia in the general U.S. population is estimated to be only around 5% [8].

A strength of this study was the inclusion of analysis based on potential risk factors for the development of anemia. Unlike previous research, this study identified sex, race, and smoking status as potential risk factors for anemia in patients with HS. This may encourage more comprehensive screening and management for HS patients at higher risk of anemia, so that both conditions may be treated simultaneously. Furthermore, physicians can feel more comfortable treating HS patients given the knowledge that certain patients will likely have comorbid anemia.

This study had several limitations. The sample size was small, making multivariate statistical analysis unreliable. Also, there was no way to produce a control group for this study because patients who would have had laboratory analysis would also have a significant dermatologic disease. It is hoped that future studies will focus on broader sampling and larger patient totals. Another goal is to determine if the severity of HS is correlated with the presence or absence of anemia in these patients as this information was not evaluated in this study.

## Conclusions

This study shows that anemia is significantly higher in this HS population than in the general U.S. population. As noted previously, more than a few of the patients in this population were evaluated for their anemias and found to be suffering from anemia of chronic disease. This might suggest that HS patients with anemia do not require a full workup to be treated appropriately for their HS. However, this bears further evaluation. Despite the limitations of this study, the elevated prevalence of anemia in the HS population should be better recognized in order to more effectively treat our patients.
